# Comparison of Macular Choroidal Thickness and Volume between Pseudoexfoliative Glaucoma and Pseudoexfoliative Syndrome

**DOI:** 10.1155/2020/8886398

**Published:** 2020-11-26

**Authors:** Fan Li, Lihua Ma, Yulei Geng, Xiaowei Yan, Hengli Zhang, Guangxian Tang, Qingli Shang

**Affiliations:** ^1^Department of Ophthalmology, Shijiazhuang People's Hospital, Shijiazhuang 050000, Hebei, China; ^2^Department of Ophthalmology, The Second Hospital of Hebei Medical University, Shijiazhuang 050000, Hebei, China

## Abstract

**Purpose:**

To evaluate the difference in macular choroidal thickness and volume between patients with pseudoexfoliative glaucoma (PXG), patients with pseudoexfoliative syndrome (PEX), and normal controls.

**Methods:**

This case-control study included 49 PXG patients (group A), 33 PEX patients (group B), and 42 sex-, age-, and axial length-matched healthy volunteer eyes (group C). The macular choroidal thickness and volume of all subjects studied were measured by enhanced depth imaging optical coherence tomography.

**Results:**

The average macular (AM) choroidal thickness was 170.79 ± 50.18 *μ*m, 184.65 ± 57.54 *μ*m, and 206.46 ± 48.90 *μ*m, and the average volume was 0.52 ± 0.15 *μ*m^3^, 0.56 ± 0.17 *μ*m^3^, and 0.63 ± 0.15 *μ*m^3^ in groups A, B, and C, respectively. The macular choroidal thickness, the volumes of various macular regions, and the average choroidal thickness and volume in group A were lower than those in group C (all *P* < 0.05). There were no significant differences in the macular choroidal thickness, volumes of various macular regions, or average choroidal thickness or volume between group A and B (all *P* > 0.05). The macular choroidal thickness and volume of the TIM and SOM in group B were lower than those in group C (*P* < 0.05). There was no association between the macular choroidal thickness of various macular regions and visual field mean defect (MD) in group A (all *P* > 0.05).

**Conclusion:**

The macular choroidal thickness in patients with PXG or PEX (TIM and SOM) is thinner than that in normal subjects. The macular choroidal thickness in patients with PXG is not significantly different from that in patients with PEX. The role of macular choroidal thickness changes in the glaucomatous damage of patients with PXG is still unclear.

## 1. Introduction

Pseudoexfoliative glaucoma (PXG) is a secondary glaucoma caused by pseudoexfoliative syndrome (PEX), accounting for approximately 25% of cases of open-angle glaucoma [1]. Exfoliative substances and pigments are deposited in the trabecular meshwork, which obstructs Schlemm's canal, resulting in the narrowing of the lumen and eventually causing the lumen wall to collapse, leading to the development of PXG [2]. PEX is an important and definitive risk factor for open-angle glaucoma. Under the same intraocular pressure, PEX eyes are more likely to develop glaucoma than non-PEX eyes [3]. Exfoliative material is not only deposited in the anterior segment tissues of the eyes, such as the corneal endothelium, lens surface, and trabecular meshwork, but may also have an impact on the posterior segment tissues of the eyes such as posterior ciliary arteries, vortex veins, and central retinal vessels [4]. Some studies [5, 6] have found hemodynamic abnormalities in retrobulbar vessels in patients with PEX and PXG.

The choroid is the vascular layer located under the retina. It has the highest perfusion rate of all blood vessels in the human body. Because of its important role in ocular blood flow, it plays an important role in the development and progression of glaucoma [6]. Choroidal thickness is proportional to its blood flow, and choroidal thickness measurement can provide important information on choroidal blood flow velocity. Measurement of choroidal thickness using enhanced-depth imaging optical coherence tomography (EDI-OCT) can provide important information on choroidal blood flow velocity [4]. At present, studies on macular choroidal thickness in eyes with PEX and PXG are controversial [7], and whether exfoliative substances can cause abnormal ocular blood flow remains unclear.

In this study, we used spectral-domain EDI-OCT to measure the thickness and volume of the choroid in the macular area of PXG eyes and PEX eyes in the Chinese population, aiming to determine the pattern of changes in the choroidal thickness of the macular area of PEX and PXG eyes.

## 2. Materials and Methods

A total of 82 patients treated in our hospital between May 2015 and May 2020 were recruited for this study. The 49 PXG patients (49 eyes) were included in group A, and 33 PEX patients (33 eyes) were included in group B. Groups A and B were all cases of monocular disease. Another 42 sex-, age-, and axial length-matched healthy volunteers (42 eyes) were included in group C. There were no significant differences in age, sex, or axial length between the three groups (Table 1).

PEX diagnostic criteria were that the characteristic features of ocular PEX could be observed under a slit-lamp microscope, such as the appearance of gray-white exfoliative material at the pupillary margin, iris surface, and anterior lens capsule; the 24-hour IOP (every 2 hours) was ≤21 mmHg; the optic disc appeared normal, and the visual field examination was normal. Diagnostic criteria for PXG included the abovementioned characteristic features of ocular PEX, IOP >21 mmHg, and glaucomatous optic nerve damage and visual field defects [8]. Diagnostic criteria for the normal control group were a normal-looking optic disc (no disc edge narrowing or optic disc hemorrhage), cup disc ratio (C/D) ≤0.3, binocular difference ≤0.2, IOP ≤21 mmHg, and normal examination of the visual field and chamber angle.

Inclusion criteria were meeting one set of the diagnostic criteria with an age of ≥50 years. Exclusion criteria were other types of glaucoma (such as closed-angle glaucoma and secondary glaucoma); history of ocular antiglaucoma medication use; previous history of ocular surgery or ocular trauma; other ophthalmic diseases, such as corneal opacity, lens opacity, or other ocular diseases affecting the examination; retinal or macular diseases of the fundus (diabetic retinopathy, retinal vein occlusion, hypertensive retinopathy, macular edema, epimacular membrane, macular degeneration, and macular hole); a diopter of spherical equivalent >±6.0 D or cylinder >±3.0 D; and systemic diseases such as diabetes and hypertension. This study followed the Helsinki Declaration and was approved by the ethics committee of Shijiazhuang People's Hospital. All subjects and their guardians signed informed consent forms.

### 2.1. Routine Examinations

All subjects underwent comprehensive eye examinations, including slit-lamp microscopy, fundus, colour fundus photograph, IOP measurement (Goldmann Applanation Tonometer), gonioscopy, vision tests, axial length measurement, and visual field examinations.

### 2.2. OCT Examinations

**Figure 1:**
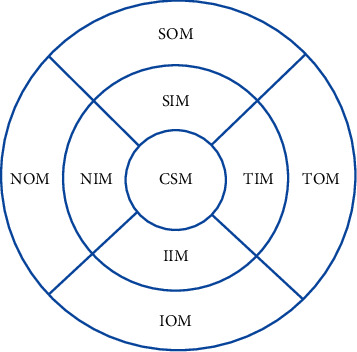
Measurement illustration of macular choroidal thickness at nine locations: CSM, central subfield macula; NIM, nasal inner macula; SIM, superior inner macula; IIM, inferior inner macula; TIM, temporal inner macula; NOM, nasal outer macula; SOM, superior outer macula; IOM, inferior outer macula; TOM, temporal outer macula.

**Figure 2:**
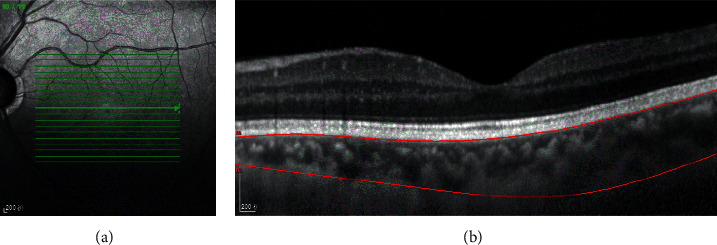
Optical coherence tomographic image (enhanced depth imaging mode) for measurement of the macular choroidal thickness.

All subjects underwent the SD-OCT (Spectralis HRA + OCT, Heidelberg, Germany). The macular thickness and volume were scanned using the EDI mode of the SD-OCT macular thickness map examination procedure. For specific measurement methods, refer to previous studies [9]. Measurement illustration of macular choroidal thickness is shown in Figure 1. On each scanned image, the inscribed segmentation line was labeled on the retinal pigment epithelium/Bruch membrane interface and the outer segmentation line was placed on the scleral/choroidal interface to represent the internal and external choroidal boundaries, as shown in Figure 2. The choroidal thickness measurements were performed by the same technician.

### 2.3. Visual Field Examination

The visual fields of all subjects were examined using the SITA-Fast 30–2 examination procedure and a Humphrey 750i visual field analyzer (Carl Zeiss, Germany). The reliability criteria included a fixation loss rate of <20%, a false negative rate of <15%, and a false positive rate of <15%. Individuals who did not meet the criteria were excluded.

### 2.4. Statistical Analysis

This is a retrospective case-control study, and all the cases are eligible for continuous selection during the study period. The data were analyzed using SPSS 19.0 statistical software. The sex composition ratio in the three groups of subjects was compared using a *χ*^2^ test. One-way ANOVA was performed for comparisons of age, axial length, visual field mean defect (MD), choroidal thickness, and volume among the three groups. An LSD-*t* test was used for pairwise comparisons. Pearson correlation analysis was used to analyze the correlation between macular thickness and visual field MD in PXG. Differences with *P* < 0.05 were considered statistically significant.

## 3. Results

The AM choroidal thickness in groups A, B, and C was 170.79 ± 50.18 *μ*m, 184.65 ± 57.54 *μ*m, and 206.46 ± 48.90 *μ*m, and the average volume was 0.52 ± 0.15 *μ*m^3^, 0.56 ± 0.17 *μ*m^3^, and 0.63 ± 0.15 *μ*m^3^, respectively. There were significant overall differences in the central subfield (CSF), nasal inner macula (NIM), superior inner macula (SIM), temporal inner macula (TIM), inferior inner macula (IIM), nasal outer macula (NOM), superior outer macula (SOM), temporal outer macula (TOM), inferior outer macula (IOM), and average macular choroidal thickness between the three groups, respectively (*F* = 5.774, *P*=0.004; *F* = 4.462, *P*=0.013; *F* = 3.658, *P*=0.029; *F* = 6.934, *P*=0.001; *F* = 5.017, *P*=0.008; *F* = 4.449, *P*=0.014; *F* = 3.407, *P*=0.036; *F* = 5.995, *P*=0.036; *F* = 4.185, *P*=0.017; *F* = 5.391, *P*=0.006), as well as in their volume (*F* = 5.469, *P*=0.005; *F* = 4.504, *P*=0.013; *F* = 3.638, *P*=0.029; *F* = 6.927, *P*=0.001; *F* = 5.121, *P*=0.007; *F* = 4.462, *P*=0.013; *F* = 4.016, *P*=0.020; *F* = 7.038, *P*=0.001; *F* = 4.221, *P*=0.017; *F* = 5.533, *P*=0.005). The macular choroidal thickness and volume of various macular regions and the average macular choroidal thickness and volume in group A were lower than those in group C (all *P* < 0.05). The macular choroidal thickness and volume of various macular regions and the average macular choroidal thickness and volume in group A were not significantly different from those in group B (all *P* > 0.05). The macular choroidal thickness and volume in the TIM and SOM in group B were lower than those in group C (*P* < 0.05). The macular choroidal thickness and volume of the CSF, NIM, SIM, IIM, NOM, TOM, and IOM and the average macular choroidal thickness and volume in group B were not significantly different from those in group C (*P* > 0.05) (Table 2). There was no association between CSF, NIM, SIM, TIM, IIM, NOM, SOM, TOM, IOM choroidal thickness, and visual field defects in group A (*r* = 0.068, *P*=0.641; *r* = 0.028, *P*=0.849; *r* = 0.129, *P*=0.376; *r* = 0.122, *P*=0.404; *r* = 0.017, *P*=0.909; *r* = 0.081, *P*=0.579; *r* = 0.057, *P*=0.697; *r* = 0.164, *P*=0.259; *r* = 0.058, *P*=0.690).

## 4. Discussion

The pathophysiology of PEX and PXG is not yet fully clear. Studies have shown that, after 10 years of follow-up, 38% of patients had progressed from monocular PEX to binocular PEX [10], 5.3% of PEX patients had progressed to PXG within 5 years, and 15.4% of PEX patients had progressed to PXG within 10 years [11]. PXG progresses rapidly, and drug treatment has a poor effect, which is why PEX develops into PXG [12]. Koz et al. [13] found that a significant proportion of PEX patients with normal IOP also experienced glaucoma changes, so they speculated that some optic disc damage in PXG eyes may not be related to intraocular pressure. A wider range of IOP fluctuations in PXG may be an important factor causing glaucoma progression, but the effect of exfoliation itself and choroidal dysfunction could not be ruled out. Detorakis et al. [5] concluded that the posterior ciliary artery of eyes with PXG had abnormal hemodynamics, the long and short posterior ciliary arteries had lower end-diastolic blood flow velocity, the resistance index in the short posterior ciliary arteries was greater, and the exfoliative material was likely to involve small vessels rather than large vessels. These pathological changes could lead to choroidal thinning [14, 15].

The results of previous studies on macular choroidal thickness in patients with PXG and PEX are controversial. The study by Dursun et al. [7] found that the choroidal thickness at the foveal and parafoveal areas (1.5 mm nasal, 3 mm nasal, 1.5 mm temporal, and 3 mm temporal to the fovea) of the macula was smaller in eyes with PXG and PEX than in normal eyes, while there was no significant difference in macular choroidal thickness between PXG eyes and PEX eyes. The study by Egrilmez et. al [16] found that the macular choroid (1.5 mm nasal, 2.5 mm nasal, 1.5 mm temporal, and 2.5 mm temporal to the fovea) was thinner in patients with pseudoexfoliative glaucoma, as compared with both healthy individuals and open-angle glaucoma patients with similar degrees of glaucomatous damage. Moghimi et al. [9] found that the macular choroid (nine locations) in PEX eyes is not significantly different from normal subjects. However, Ozge et al. [17] concluded that there was no significant difference in choroidal thickness in the foveal or parafoveal regions between PXG eyes, PEX eyes, and normal eyes. A study by Bayhan et al. [4] found that the choroidal thickness at 3 mm nasal to the fovea (the part of macula that is closest to the optic nerve head) was significantly smaller in PXG eyes than in normal eyes.

This study found that the mean macular choroidal thickness was 170.79 ± 50.18 *μ*m, 184.65 ± 57.54 *μ*m, and 206.46 ± 48.90 *μ*m in the PXG, PEX, and normal eyes, respectively. The distribution pattern of macular choroidal thickness in all three groups was inner macula > outer macula. The distribution pattern of the inner macula in the PXG group and PEX group was SIM > TIM > IIM > NIM, and the pattern of the outer macula was SOM > TOM > IOM > NOM. In the normal eye group, the distribution pattern of the inner macula was TIM > SIM > IIM > NIM and the pattern of the outer macula was SOM > TOM > IOM > NOM. The choroidal thickness in each macular area was thinner in both PXG and PEX eyes than in normal eyes, so we can speculate that, during the progression of PXG, the macular choroidal thickness gradually thins and the thinning rate in the temporal inner macula is faster. Even in PEX eyes with normal intraocular pressure, their macular choroidal thickness was thinner than that in normal eyes, and the reason may be that PEX caused ischemic disorder [18]. This study found that macular choroidal thickness was slightly but not significantly thinner in PXG eyes than in PEX eyes, further demonstrating that PXG is a disease characterized by ocular hypertension. However, PXG may have risk factors unrelated to intraocular pressure, such as ocular and retrobulbar blood flow disorders [19]. Both this study and the study by Dursun et al. [7] confirm that the macular choroidal thickness in PXG eyes and PEX eyes is thinner than that in normal eyes, though the mode of subdivision of the macular choroid and the measurement locations were slightly different between the two studies.

This study has some limitations. The first limitation was the relatively small number of patients in the study groups. We set strict inclusion criteria to ensure that the observation group and the control group had matched parameters, such as age, gender, and axial length to reduce interference from individual variations that might affect the results to some extent. The second limitation was a lack of automatic measurement software, which may have resulted in some error in our results. In addition, the choroid is a highly dynamic vascular tissue and simple measurement of choroidal thickness cannot sufficiently describe the hemodynamic and physiological changes observed in ocular diseases.

## 5. Conclusion

The macular choroidal thickness in patients with PXG or PEX (TIM and SOM) is thinner than that in normal subjects. The macular choroidal thickness in patients with PXG is not significantly different from that in patients with PEX. Therefore, large-scale multicenter studies are needed to investigate the effect of change in choroidal thickness on the development of glaucoma in the cases with PEX.

## Figures and Tables

**Table 1:** Baseline characteristics of the study groups.

Groups	Eyes (*n*)	Gender (*n*) M/F	Age (years)	IOP (mmHg)	AL (mm)	MD (dB)
Group A	49	25/24	75.61 ± 6.58 (62–88)	28.00 ± 9.74 (12–50)	23.17 ± 0.99	−16.65 ± 9.60
Group B	33	16/17	75.94 ± 7.89 (56–89)	16.39 ± 2.79 (11–21)	23.27 ± 0.70	−1.22 ± 0.34
Group C	42	20/22	75.07 ± 7.37 (56–86)	15.31 ± 2.55 (11–21)	23.29 ± 0.86	−1.14 ± 0.40
*χ* ^2^ */F*		0.114	0.141	52.962	0.248	96.814
*P* value		0.945	0.869	≤0.001	0.781	≤0.001

M, male; F, female; IOP, intraocular pressure; AL, axial length; MD, mean defect.

**Table 2:** Comparisons of macular choroidal thickness by EDI-OCT in three groups.

Regions		Group A	Group B	Group C	*P* value A-B	*P* value A-C	*P* value B-C
CSM	TH, *μ*m	182.53 ± 56.38	198.58 ± 62.06	223.31 ± 54.15	0.215	0.001^※^	0.066
	V, *μ*m^3^	0.14 ± 0.05	0.16 ± 0.05	0.18 ± 0.04	0.207	0.001^※^	0.082

NIM	TH, *μ*m	163.18 ± 54.26	179.21 ± 61.84	199.07 ± 56.59	0.215	0.003^※^	0.138
	V, *μ*m^3^	0.26 ± 0.09	0.28 ± 0.10	0.31 ± 0.09	0.210	0.003^※^	0.138

SIM	TH, *μ*m	190.61 ± 59.21	196.42 ± 62.31	222.36 ± 53.13	0.658	0.011^※^	0.057
	V, *μ*m^3^	0.30 ± 0.09	0.31 ± 0.10	0.35 ± 0.08	0.617	0.010^※^	0.064

TIM	TH, *μ*m	181.27 ± 55.34	195.79 ± 59.36	224.52 ± 53.13	0.249	≤0.001^※^	0.028^※^
	V, *μ*m^3^	0.28 ± 0.09	0.31 ± 0.09	0.35 ± 0.08	0.238	≤0.001^※^	0.030^※^

IIM	TH, *μ*m	168.39 ± 58.93	188.94 ± 64.37	207.36 ± 53.38	0.122	0.002^※^	0.179
	V, *μ*m^3^	0.26 ± 0.09	0.30 ± 0.10	0.33 ± 0.08	0.121	0.002^※^	0.172

NOM	TH, *μ*m	131.22 ± 44.69	145.94 ± 58.47	164.93 ± 59.34	0.226	0.003^※^	0.131
	V, *μ*m^3^	0.70 ± 0.24	0.77 ± 0.31	0.87 ± 0.32	0.227	0.003^※^	0.130

SOM	TH, *μ*m	190.08 ± 54.72	191.61 ± 59.64	217.88 ± 51.27	0.902	0.018^※^	0.042^※^
	V, *μ*m^3^	1.00 ± 0.29	0.99 ± 0.33	1.16 ± 0.27	0.777	0.017^※^	0.015^※^

TOM	TH, *μ*m	168.20 ± 46.35	184.42 ± 50.78	203.43 ± 48.78	0.139	0.001^※^	0.094
	V, *μ*m^3^	0.87 ± 0.25	0.98 ± 0.27	1.08 ± 0.26	0.078	≤0.001^※^	0.099

IOM	TH, *μ*m	161.61 ± 54.36	180.97 ± 59.19	195.31 ± 54.63	0.126	0.005^※^	0.271
	V, *μ*m^3^	0.86 ± 0.29	0.96 ± 0.31	1.04 ± 0.29	0.122	0.005^※^	0.274

AM	TH, *μ*m	170.79 ± 50.18	184.65 ± 57.54	206.46 ± 48.90	0.237	0.001^※^	0.073
	V, *μ*m^3^	0.52 ± 0.15	0.56 ± 0.17	0.63 ± 0.15	0.244	0.001^※^	0.065

CSM, central subﬁeld macula; NIM, nasal inner macula; SIM, superior inner macula; IIM, inferior inner macula; TIM, temporal inner macula; NOM, nasal outer macula; SOM, superior outer macula; IOM, inferior outer macula; TOM, temporal outer macula; AM, average macula; TH, thickness; V, volume. Data are expressed as means ± standard deviation. ^※^*P* < 0.05.

## Data Availability

The data used to support the findings of this study are available from the corresponding author upon request.
